# Testosterone-like immunoreactivity in hair measured in minute sample amounts - a competitive radioimmunoassay with an adequate limit of detection

**DOI:** 10.1038/s41598-017-17930-w

**Published:** 2017-12-15

**Authors:** Julia K. Slezak, Jakob O. Ström, Elvar Theodorsson

**Affiliations:** 1 Department of Clinical Chemistry and Department of Clinical and Experimental Medicine, Linköping University, Linköping, Sweden; 2 Department of Neurology, Faculty of Medicine and Health, Örebro University, Örebro, Sweden

## Abstract

The concentrations of testosterone deposited in hair during hair growth may provide a retrospective reflection of the concentrations of bioactive testosterone in plasma. The objective of this study was to develop a radioimmunoassay with a sufficiently low limit of detection to measure the testosterone-like immunoreactivity in smaller hair samples (5 mg) than used in earlier studies, and to compare three different extraction procedures. The competitive radioimmunoassay consisted of a polyclonal antiserum (immunogen testosterone-7α-BSA) and a radioligand synthesised from testosterone-3-CMO-histamine. The within-assay and total coefficients of variation in the working range was 3% and 4.5%, respectively. The limit of detection was 0.87 pg/mL, which is equivalent to 0.12 pg/mg testosterone in 5 mg of hair. The concentration of testosterone-like immunoreactivity in hair samples was 1.23 (SD 0.47) pg/mg in women and 2.67 (SD 0.58) pg/mg in men (pulverised hair). Significantly improved precision was found when pulverised hair was used compared to non-pulverised hair. Our data indicate that pulverisation of the hair prior to hormone extraction is crucial. Detection limits fit for the intended purpose are achievable with 5 mg samples of hair.

## Introduction

The main biological effects of sex hormones are dependent on their long term concentrations in the tissues, which is one of the reasons why repeated blood sampling is a prerequisite for diagnosing hypogonadism^1^. Sex hormones may also be measured in saliva and in 24 hour collections of urine. However, 24 hour collections of urine are cumbersome and fraught with well-known risks of incomplete collections. The predominant disadvantage of analysing hormones in serum/plasma, saliva or urine is the pronounced intra- and interday variability in hormone levels, including the levels of binding proteins, which makes current methods unsuited for evaluation of long-term hormone levels^2^. The concentrations of steroid hormones deposited in hair are retrospective reflections of the biologically active plasma concentrations of the hormones during the period of growth. Scalp hair grows approximately 1 cm/month which enables a timed retrospective evaluation of the long term hormone levels using only one sample^3^. Hormone analysis in hair has the additional advantage of circumventing difficulties in determination of the biologically active fraction, as well as being stable at room temperature for months and years facilitating storage and transport^4^.

The field of hair hormone analysis has so far primarily focused on the assessment of chronic stress and analysis of the stress hormone cortisol in hair. Thus, the clinical value of hair testosterone measurement is yet to be determined. Meanwhile, the support for hair cortisol analysis in different clinical settings is steadily increasing as increased cortisol levels in hair have been found in patients with hypercortisolism, in shift workers and in unemployed persons^5–7^. However, the attempts to validate hormone concentrations in hair using, for example, repeated saliva samplings, have been challenging. The significant correlation between cortisol measured in hair and in saliva in humans has been, at best, r = 0.61 (90 saliva samples) and r = 0.57 (18 saliva samples)^8,9^. The magnitude of this association between saliva and hair cortisol raises the question whether other factors contribute to the hormone concentrations found in hair, such as local hormone conversion and/or production in the skin (and its appendages), or inherent genetic differences between individuals^10,11^.

Even though hair sampling is non-invasive and produces a minimal discomfort, it is desirable to minimise the amount of sample required because of the potential negative cosmetic effect for the people donating a sample. Using minute hair amounts, such as 5 mg hair per sample, calls for analytical methods with sufficiently low detection limits. The methodological shortcomings of sex steroid assays have quite recently been discussed^12,13^. There are well known issues with selectivity and insufficient limits of detection. However, it is still possible to achieve adequate precision and limit of detection with validated immunoassay methods including the radioimmunoassay (RIA)^14,15^. Liquid chromatography tandem-mass spectrometry (LC-MS/MS) has the advantage of high selectivity, but there are reported problems with the limit of quantification requiring large amounts of sample, as well as interferences from the hair matrix^16^. Also, in spectrophotometric methods the hair colour may have undesirable effects on the detection. We chose the competitive RIA in order to achieve a sufficiently low limit of detection and to avoid interferences such as those mentioned above. The RIA developed in the present study enables measurement of testosterone-like immunoreactive molecules in 5 mg samples of hair – a smaller sample amount than earlier reported.

In the current study we examine the effects of sex and the disintegration of the hair (during the extraction process) on the concentrations of testosterone-like immunoreactivity in hair. The rationale for this is that we expect the biological difference between men and women to overshadow any other physiological or circumstantial sources of variation that could affect the androgenic hormone concentrations in hair. Choosing an anti-testosterone antiserum with <0.1% cross-reactivity for cortisol, 17β-estradiol and progesterone we have strived to design a RIA that predominantly measures testosterone, but also enables measurement of other testosterone-like molecules. We also hypothesize that the disintegration of hair during the extraction procedure is important, in contrast to the current trend in the field which is to use non-pulverised hair. The objective of this study was to develop a RIA with a sufficiently low limit of detection to measure the testosterone-like immunoreactivity in both women and men, in smaller hair sample amounts (5 mg) than previously used, and to compare three different hair extraction procedures. Our primary aim when comparing extraction procedures was to explore the effect on the measurement precision expressed as the relative standard deviation (RSD) of triplicate samples from each individual.

## Materials and Methods

### Chemicals and assay constituents

The gamma radiation from ^125^I represents the primary safety hazard posed by the current RIA method. The very low concentrations of the radioligand used in the RIA in order to obtain the low detection limit means that the gamma radiation from performing the immunoassays itself is not significantly different from the background radiation. However, national and local safety regulations need to be carefully adhered to when labelling and purifying the radioligand. For the competitive RIA a polyclonal rabbit antiserum was used (Immunogen testosterone-7α-BSA, RRID:AB_261672, T4276, lot 80K4843, Sigma Aldrich, St. Louis, MO). The cross-reactivity of the antiserum was 23.0, 1.5, 0.2 and 1.7% for 5α-dihydrotestosterone, 17α-epitestosterone, dehydroepiandrosterone and androstenedione, respectively. The radioligand was ^125^I (PerkinElmer, Stockholm, Sweden) labelled histamine-derived testosterone (testosterone-3-CMO-histamine, 21505-1, Research Plus Inc., Barnegat, NJ) prepared using the chloramine-T method. The reaction product was purified on C_18_ – reverse-phase high performance liquid chromatography (HPLC) column using a gradient of 20–50% acetonitrile with 0.1% trifluoroacetic acid. The RIA was composed of the radioligand diluted in incubation buffer (approximately 7000 cpm/0.1 mL) and a dilution of the antiserum resulting in 30–50% binding of the radioligand, after incubation overnight. The calibrator (testosterone, T5411, lot 081M8702, Sigma Aldrich, Saint Louis, MO) was verified with a European pharmacopoeia reference standard (testosterone, T0100000, batch 2, EDQM, Strasbourg, France). The incubation buffer was a 0.05 M phosphate buffer, pH 7.4, 0.2% bovine serum albumin, 0.1% Triton X-100 and 0.02% sodium azide.

**Figure 1:**
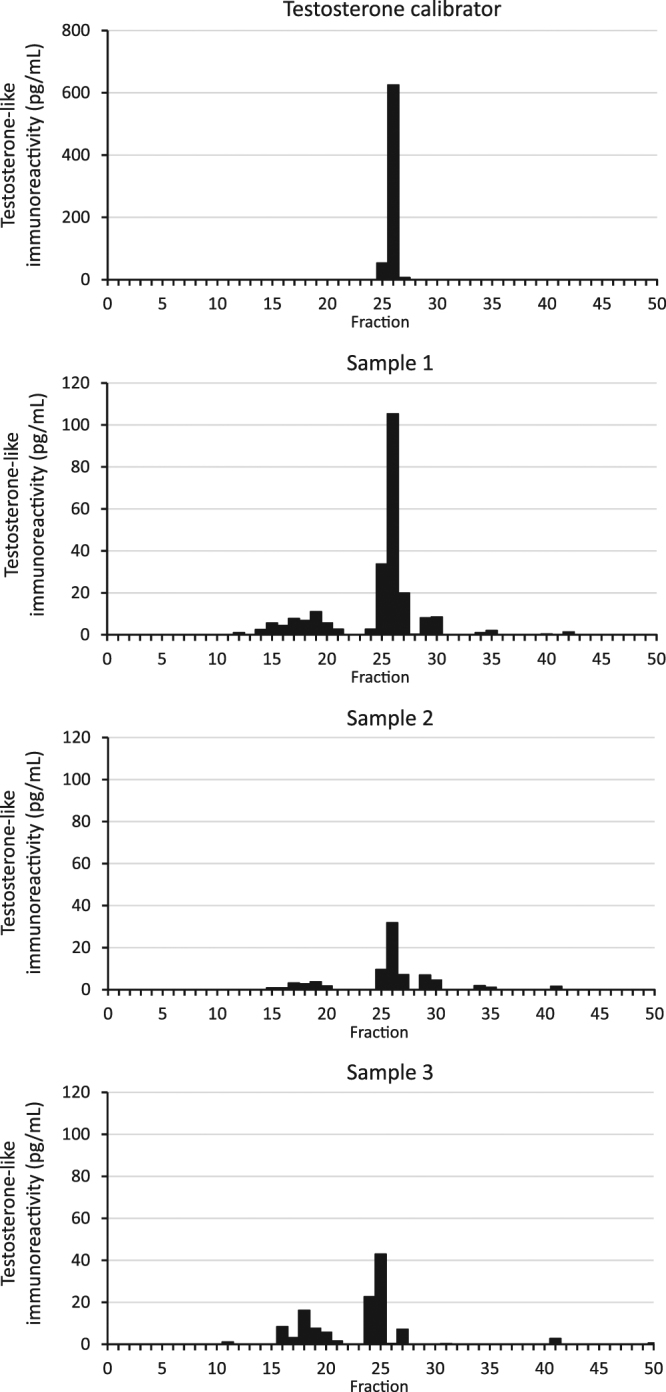
Reverse-phase HPLC analysis of the testosterone-like immunoreactivity measured in methanol extracts of pulverised hair from three males. The immunoreactive component corresponding to testosterone represents about half of the measured immunoreactivity.

The testosterone-like immunoreactivity measured in the present study consisted primarily of testosterone, but also other immunoreactive components, as characterized in a reverse-phase HPLC on hair samples from three males shown in Fig. 1.

### Analytical and validation parameters

**Figure 2:**
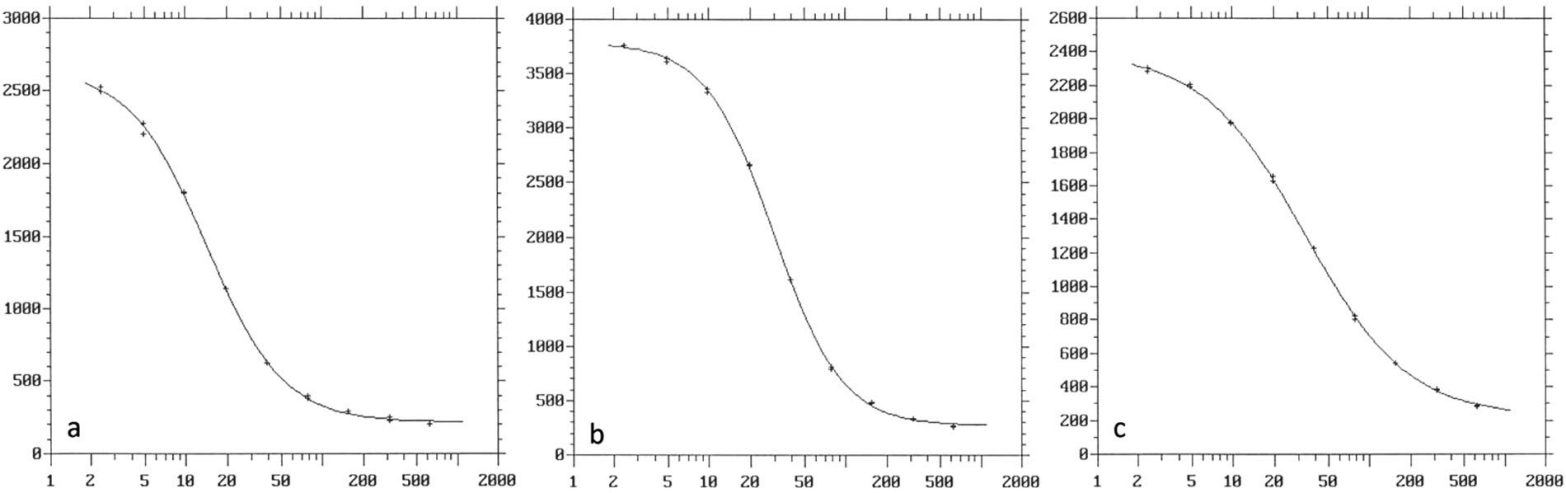
Examples of three calibration curves. Units are CPM on the y-axis and pg/mL testosterone on the x-axis. The calibrator range is 2.4–625 pg/mL. Curve A has an optimal antibody-to-radioligand binding of 43% compared to 56% in curve B, which has displaced curve B slightly to the right with an inferior detection limit as a consequence. Another factor influencing analytical sensitivity (slope of the dose-response curve) is the gradual decomposition of the radioligand. Curve C is one of the calibrators used in the variance component analysis in this paper.

The calibrator range was 2.4–625 pg/mL which translates to 0.34–89 pg/mg testosterone when 5 mg of hair per sample is used (see Fig. 2). The precision of the assay was determined through repeated measurement of ten replicates. No hair samples deprived of androgens were available thus testosterone was diluted in the analytical buffer to concentrations of 10, 45 and 90 pg/mL testosterone. The within-assay and between-assay variability at 10, 45 and 90 pg/mL was 9.6%, 3.0%, 2.9%, and 39.8%, 11.8%, 10.9%, respectively. The total variability at 10, 45 and 90 pg/mL was 15%, 4.5% and 4.3%. The fitted equation to the working range was y = 0.95x − 0.68, R^2^ = 0.99. The limit of detection (LOD) was 0.87 pg/mL, which is below the range of the calibrator. The limit of quantification (LOQ) is commonly defined as the lowest concentration that can be measured with a specific RSD. In the field of masspectrometric hormone analysis the LOQ is usually defined as the analyte concentration measured with an RSD of 15 or 20%^16^. The lowest concentration in our working range was measured with an intra-assay RSD slightly below 10%^17^.

### Study population and hair sampling

The study protocol was approved by the Regional Ethical Review Board of Linköping (2013/100–31) before participant recruitment. The study was performed in accordance with relevant guidelines and regulations. Informed consent was obtained from all participants. All individuals were older than 18 years. Aiming at detecting a significant difference in hair testosterone between men and women with the risk of a β-error of 0.8, we used rounded values from the study by Gao *et al*. and determined that approximately sixteen individuals would suffice^18^. Because of the unknown effect of the different extraction methods on the analysis precision, we chose to include eleven women and nine men. Hair samples were collected from the posterior vertex. A strand of hair was fixated between two sheets of Parafilm using forceps and then cut off as close to the scalp as possible. The Parafilm facilitated measurement and marking of the most proximal hair segment that was 3 cm long (using an ordinary ruler), and it was removed as the hair was cut into small pieces of approximately 2 to 3 mm length and transferred into Eppendorf tubes. Each hair sample was divided into multiple replicates, three replicates from each individual was used in each extraction procedure. Mean weight for the sample replicates was 5.3 mg (SD 0.20) and 5.1 mg (SD 0.27) for women and men, respectively. The samples were stored in room temperature until extraction.

### Extraction procedures

A total of three extraction procedures were evaluated in which the fragmentation of the hair samples varied, see Table 1 for details. Methanol was used to extract the testosterone. In the first extraction procedure (extraction A), testosterone was extracted directly from the finely cut hair. In the second extraction procedure (extraction B), the hair was pulverised using a ball mill (Retch TissueLyser II, Qiagen, Hilden, Germany) prior to the extraction of testosterone. In the third extraction procedure (extraction C) sodium hydroxide was used to disintegrate the hair completely, hydrochloric acid was used for neutralization, and the testosterone was subsequently extracted. During the extraction with methanol, the tubes were fixated in metal holders on an oblique plate on a horizontal shaker so that the steel balls slowly oscillated inside the tubes. After incubation for 20 to 24 hours, the samples were centrifuged and the supernatant was transferred to new sample tubes for evaporation in Savant SpeedVac Plus SC210 A with a cold trap using Edwards XDS 5 vacuum pump. The dry extracts were stored at 4 °C until analysis.

**Table 1:** Extraction procedures.

	Extraction A	Extraction B	Extraction C
Preparation		A 5 mm steel ball was put in each sample tube. The tubes were put in aluminium holders (especially constructed for this purpose) and submerged in liquid nitrogen for 2 minutes.	
Disintegration		The sample tubes were quickly transferred to the ball mill and grinded for 2 min at 23 Hz.	The samples were incubated with 100 µL 1 M NaOH at 95 °C for 10 min. When the tubes recovered to RT 100 µL 1 M HCl was added.
Incubation with methanol	1 mL of MeOH was added to each sample. The tubes were incubated in RT on a shaker for 24 hours.	1 mL of MeOH was added to each sample. The tubes were incubated in RT on a shaker for 20 hours.	1 mL of MeOH was added to each sample. The tubes were incubated in RT on a shaker for 20 hours.
Centrifugation and evaporation	The samples were centrifuged at 1922 g for 10 min, 700 µL of the supernatant was transferred to new tubes and the methanol evaporated.	The samples were centrifuged at 1922 g for 10 min, 700 µL of the supernatant was transferred to new tubes and the methanol evaporated.	The samples were centrifuged at 1922 g for 10 min, 700 µL of the supernatant was transferred to new tubes and the methanol evaporated.

Abbreviations: MeOH, methanol; RT, room temperature; NaOH, sodium hydroxide; HCl, hydrochloric acid.

### Radioimmunoassay

All samples were analysed in the same assay. The dry extracts were dissolved in incubation buffer (0.5 mL). All sample replicates were analysed in duplicate. The antiserum, tracer and sample were added at equal volumes (0.1 mL) and the assay was incubated at 4 °C for approximately 16 hours. Separation was performed with solid-phase coupled anti-rabbit IgG (Sac-Cel, AA-SAC1, IDS Ltd, Boldon, England), 75 µL per tube was added and the tubes were incubated for 30 min in room temperature. Distilled water (2 mL) was added to each sample before centrifugation at 1811 g for 15 min at 4 °C after which the supernatant was poured off. The bound fraction was counted for 8 minutes using the 2470 WIZARD^2^ Automatic gamma counter (Perkin-Elmer, Waltham, MA).

### Reverse-phase HPLC

Reverse-phase HPLC was performed using Jones Genesis^®^ 4 µm, 120 Å, 4.6 mm × 250 mm column eluted with a 40 min linear gradient of acetonitrile in water containing 0.1% trifluoroacetic acid (see Fig. 1). Two Waters Model 515 pumps (www.waters.com) were controlled by a Clarity chromatography software (www.dataapex.com). A gradient of 20–100% acetonitrile was used. Samples were passed through Millipore GS filters (0.22 µm) before chromatography to remove particulate matter. Fractions of 1.0 mL were collected at an elution rate of 1.0 mL/min, a total of 50 fractions, the last 10 fractions were eluted with 100% acetonitrile. Each fraction was lyophilized and re-dissolved in 0.1 mL RIA buffer before analysis. The fractions were assayed for immunoreactivity in the tubes used for their collection.

### Statistical analysis

Level of significance for all statistical analyses was set at α = 0.05, the tests were two-sided. The distribution of hair testosterone-like immunoreactivity in both men and women was positively skewed. The difference in mean hair testosterone-like immunoreactivity between the sexes (within each extraction method) was explored using the Mann-Whitney U test applied to the averaged testosterone-like immunoreactivity from the triplicates in each individual. The effect of the different extraction procedures on the precision was explored by calculating the RSD for each individual’s triplicates. Taking into account that the testosterone-like immunoreactivity of the same 20 individuals was measured in each extraction method, the related-samples Wilcoxon Signed Rank Test was chosen. Further, we used the ROC statistics and the area under the ROC curve (AUC) to explore if the different extraction procedures had any significant effect on the ability to discriminate between the sexes. IBM SPSS Statistics version 23.0 was used for the statistical analysis mentioned above. Stata version 14 was used to calculate the difference between the methods’ AUC using the chi2-test. Considering the risk of low analyte concentrations in women we explored the distribution of raw testosterone-like immunoreactivity levels (pg/mL testosterone-like immunoreactivity, before adjustment for sample weight) compared to the RSD of triplicates. 90% and 99% confidence intervals were calculated where applicable.

## Results

The testosterone-like immunoreactivity was significantly higher (extraction A p = 0.007, extraction B p = 0.000) in hair samples from men compared to women, irrespective of the extraction method, see Table 2. Results from the third extraction procedure (extraction C) are not shown because a subsequent control of pH in each sample revealed a skew towards more acidic pH rendering the results unreliable. Mean pH in the samples from extraction C was 6.6 (SD 0.05) while the analysis buffer had a pH of 7.6 (SD 0.04). There was no evident effect on the recovery, i.e. mean testosterone-like immunoreactivity in each individual, between the extraction methods (n_A_ = 20, n_B_ = 20, p = 0.313, related-samples Wilcoxon signed rank test).

**Table 2:** Testosterone-like immunoreactivity in hair.

	Sex	n	Mean	SD	Median	99% CI	p-value
Extraction A	Men	9	2.67	0.62	2.63	1.97–3.37	0.007
	Women	11	1.62	1.06	1.38	0.60–2.64	
Extraction B	Men	9	2.67	0.58	2.41	2.01–3.32	0.000
	Women	11	1.23	0.47	1.07	0.79–1.68	

The unit is pg/mg. Extraction A used non-pulverised hair, extraction B used pulverised hair. The descriptive statistics were calculated on the mean testosterone-like immunoreactivity levels from the triplicates in each individual. There was a significant difference in hair mean testosterone-like immunoreactivity concentrations between men and women irrespective of the extraction method (Mann-Whitney U test, n = 20, p = 0.007 resp 0.000). Note that the 99% CI for men compared to women does not overlap in extraction B. There was no substantial change to the confidence intervals when they were re-calculated using log-transformed values.

**Figure 3:**
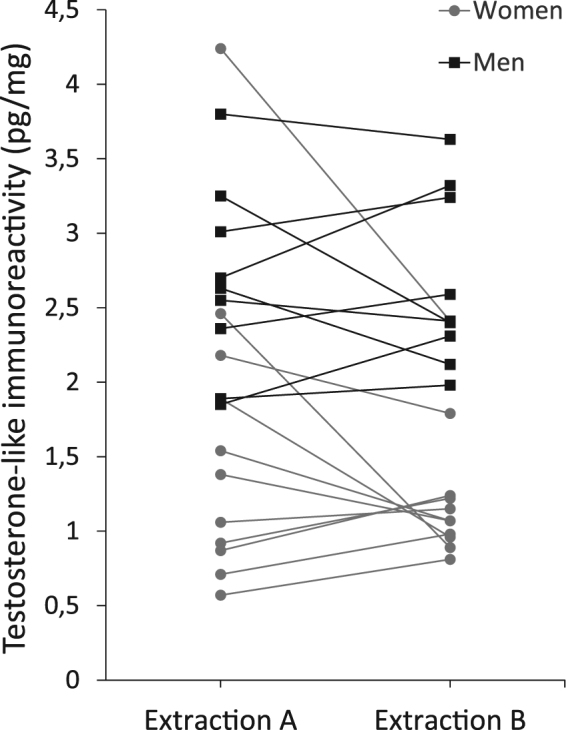
Testosterone-like immunoreactivity in the hair of eleven women and nine men. In both extraction methods the same hair samples were analysed in triplicates. The values shown in the graph are the mean hair testosterone-like immunoreactivity concentrations, from both extraction methods, in each individual.

As shown in Fig. 3, the testosterone-like immunoreactivity in the hair of men and women overlaps slightly. Examining the 99% confidence intervals (see Table 2) suggested that using pulverised hair for hormone extraction produced a better separation between the sexes. This notion was supported by the diagnostic properties of the ROC curves using our hair data to predict the sex. We explored the ROC statistics in two ways, first we compared the extraction methods using averaged hair testosterone-like immunoreactivity from the 20 individuals in the study (n_A_ = 20, n_B_ = 20). This showed a trend (p = 0.071) towards a better model when pulverised hair was used, see Table 3. To explore the effect of the number of observations we then treated each concentration as an independent sample (n_A_ = 59, n_B_ = 59, one replicate in extraction A was lost during the measurement) which revealed a high significance (p = 0.001) favouring pulverised hair to find the best distinction between the sexes.

**Table 3:** The ROC statistics comparing extraction methods.

		n	ROC area	Standard error	90% CI	p-value
Averaged concentrations	Extraction A	20	0.843	0.098	0.686–1.000	0.071
	Extraction B	20	0.956	0.048	0.876–1.000	
Independent sample concentrations	Extraction A	59	0.828	0.057	0.734–0.921	0.001
	Extraction B	59	0.963	0.023	0.925–1.000	

One of the replicates for a male in extraction A was lost during analysis reducing the total number of observations per extraction method to 59. A cut-off value of 1.9 pg/mg was associated with a sensitivity of 96% and a specificity of 91% when pulverised hair was used for analysis, the same cut-off value was associated with a sensitivity of 85% and a specificity of 73% when non-pulverised hair was used (n = 59). Calculating the ROC statistics using only mean hair testosterone-like immunoreactivity concentrations showed a trend towards better separation between the sexes favouring pulverised hair (n = 20, p = 0.071).

**Figure 4:**
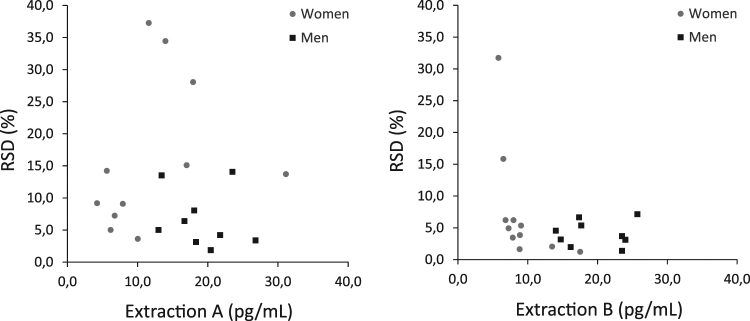
Mean raw testosterone-like immunoreactivity concentrations (pg/mL), n = 20. To each raw measurement an RSD is presented, and it is the RSD from each individual’s triplicates (calculated in the unit pg/mg). None of the men’s raw measurements fell below the lower end of the working range (10 pg/mL), where the intra-assay variability was about 10%. In pulverised hair (extraction B) it is shown that the majority of all measurements have an RSD below 10%, including those below 10 pg/mL.

Exploring the RSD of the triplicates with the related-samples Wilcoxon Signed Rank Test showed that the RSD was significantly larger (n_A_ = 20, n_B_ = 20, p = 0.009) when the testosterone-like immunoreactivity was extracted from non-pulverised hair (extraction A) compared to pulverised hair (extraction B). In extraction B the mean RSD was 6.0% (median RSD 4.5%, SD 6.9), while in extraction A the mean RSD was 12% (median RSD 8.5%, SD 10). Some of the raw measurements (pg/mL) of testosterone-like immunoreactivity in women were below 10 pg/mL, the lowest concentration in the working range, see Fig. 4. None of the men’s raw measurements fell outside the working range of the RIA. The scatter plots indicate that most measurements below 10 pg/mL were not associated with a greater RSD.

Reverse-phase HPLC analysis of the testosterone-like immunoreactivity measured in methanol extracts of pulverised hair (see Fig. 1) showed that it consisted of five immunoreactive components, three of them more lipophilic than testosterone. The immunoreactive component corresponding to testosterone represented about half of the measured immunoreactivity.

## Discussion

Using an immunoassay to measure testosterone-like immunoreactivity in hair has enabled measurement of very low concentrations in hair samples from both men and women. A significant difference between men and women in the concentration of testosterone-like immunoreactivity concentrations was found, indicating that the testosterone-like immunoreactivity reflects a true biological difference in androgenic hormone concentrations between males and females. The range of the testosterone-like immunoreactivity measured in hair overlapped slightly between men and women (see Fig. 3) which is consistent with previous research, including masspectrometric methods specific for hair testosterone^18–20^. The present results indicate that the choice of extraction method has a significant effect on the measurement precision. The smallest imprecision and the best separation between the sexes could be found in the samples extracted from pulverised hair (extraction B). Further, the overlap in the concentrations of testosterone-like immunoreactivity in hair between men and women suggests that it is important to choose a measurement method with a low imprecision to be able to detect significant differences between individuals and to monitor changes in concentrations in individuals over time. The choice of RIA as a measurement method has enabled measurement of testosterone-like immunoreactivity in smaller hair samples than previously reported.

The first study of endogenous testosterone in hair was published in 1997^21^. Until now, the methods have developed with decreasing sample amounts due to improved detection limits and simplified extraction procedures going from chemical disintegration with sodium hydroxide to extracting hormones from intact pieces of hair, see Table 4. The effect of pulverisation versus finely cut hair on hair testosterone in humans was previously evaluated by Gao *et al*.^18^, stating that the pulverisation was superfluous since the ratio between whole hair and pulverised hair ranged between 77 to 104%. There was no information regarding the potential effect of the extraction procedures on measurement precision. A similar comparison of extraction methods was performed by Davenport *et al*., analysing cortisol in rhesus macaques, with lesser variability in hair samples that were powdered as well as a greater recovery of cortisol^22^. In the current study, the extraction methods did not differ significantly regarding recovery. The hair samples extracted without pulverisation (extraction A) were incubated with methanol slightly longer than the pulverised samples (extraction B). The reason for that was to equate the time spent on all extraction methods and to enable the most complete possible extraction from the non-pulverised samples, which ultimately did not prove to be superior to pulverised hair. It can be speculated that a lesser variability could be achieved if the incubation time would be extended for the non-pulverised samples. However, that would prove extraction B to be more time efficient. Last but not least, an undesirable effect of pulverisation was mentioned by Gaudl *et al*. where homogenization of hair samples with a ball mill resulted in worsened reproducibility and there was no benefit in signal/noise ratios^23^. Worth pointing out is that in our extraction protocol cooling of the samples in liquid nitrogen prevents a negative effect of heat during the homogenization and it ameliorates the pulverisation by making the hairs more brittle.

**Table 4:** Compilation of published methods for hair testosterone analysis between the years 1997 and 2015.

Ref	Extraction	Analysis	Hair testosterone concentration (pg/mg)					
			Men		Women		Children	
			Mean ± SD	Median (range)	Mean ± SD	Median (range)	Mean ± SD	Median (range)
21	P	EIA	3.8 ± 0.5				1.5 ± 0.5	
31	S	RIA and GC-MS	9.89 ± 6.0	(3.72–22.41)	1.21 ± 0.89	(ND − 3.12)	1.46 ± 0.58	
20	P	GC-MS	3.0 ± 0.6	2.7 (2.5–4.2)	1.9 ± 0.9	1.7 (1.0–3.4)		
32	W	RIA	5.88					
33	S	GC-MS	3.8	3.1 (1.2–11.4)				
34	S	GC-MS	2.7	2.0 (0.5–9.8)		0.6 (ND − 2.4)		
35	P	RIA	10.7	(3.6–23.3)	3.6	(1.7–6.4)	1.7	(0.6–2.7)
36	S	GC-MS	9.12 ± 5.71	8.35 (0.39–18.6)				
37	S	GC-MS	11.0 ± 2.7	(6.8–17.5)				
28	W	ELISA	1.85^a^	(0.58–3.07)^a^				
38	S	GC-MS/MS	7.4^b^	6.0 (0.8–24.2)^b^	5.3^b^	3.1 (0.1–16.8)^b^	2.6^b^	1.4 (0.2–11.5)^b^
39	S	LC-MS/MS	2.67	1.98 (0.7–11.81)	1.62	1.03 (0.33–6.05)		
18	W	LC-MS/MS	1.96 ± 0.74	(1.16–4.18)	1.00 ± 0.56	(0.64–2.82)		
19	W	LC-MS/MS	1.8 ± 1.01		1.2 ± 0.38			
40	W	ELISA	5.17	(2.39–24.64)				
41	W	ELISA	8.0^c^ 5.5^d^	4.2–11^c^ 2.7–6.7^d^				
26	W	LC-MS/MS	?^e^					

Abbreviations: S, sodium hydroxide digestion; P, pulverisation; W, whole hair (cut in small pieces); EIA, enzyme immunoassay; ELISA, enzyme-linked immunosorbent assay; RIA, radioimmunoassay; GC-MS, gas chromatography mass spectrometry; LC-MS/MS, liquid chromatography tandem mass spectrometry.

Notes: ^a^pg/g. ^b^Statistically significant difference only between men and children. ^c^Men ≤ 50 years old. ^d^Men > 50 years old. ^e^Not specified, limit of quantification is 2.3 pg/mg for a 10 mg hair sample.

Collecting a lock of hair with a sample weight of 5 (to 10 mg) per centimetre will result in a hairless spot on the scalp of approximately 5 × 15 mm^2^. When studying monthly variations in hair testosterone it is needless to say that a sample weight of, for example, 30 mg per centimetre is not feasible. The sources of variability that are included in the RSD of triplicates are, theoretically, the intra-assay variability, the variability associated with the hair matrix, and also the potential variation of hormone concentrations within the lock of hair. There is a random distribution in the growth phases in hair follicles (anagen phase compared to the telogen phase). Minimising sample weights will increase the theoretical risk of collecting a non-representative sample since the growth of the hair varies due to location on the scalp^24^ and the season^25^. We believe that keeping sample weights at 5 mg is a reasonable minimum to avoid increased confounding from minor variables such as those mentioned above. Furthermore, the women’s raw hormone concentrations were at the lower end of the working range (see Fig. 4). To avoid unnecessary variability in future analyses we recommend that hair samples from females, or hypogonadal males, should weigh 10 mg.

We have excluded the washing procedure prior to extraction in all three extraction methods we compared. The washing procedure has been repeatedly discussed throughout the years, most recently by Noppe *et al*.^26^, raising concern about the risk of leaching endogenous steroids from within the hair shaft, especially in the most distal segments. Since the risk of external contamination with testosterone is reasonably much lower than the risk of contamination with topical cortisone when analysing cortisol in hair, we have chosen to skip the washing procedure.

There is an ongoing debate regarding testosterone levels in aging males, and the effects of testosterone concentrations on morbidity and mortality. Even if some evidence supports the beneficial effects of testosterone on, for example, body composition^27^, there is yet no consensus on the long-term effects of testosterone replacement therapy on the cardio- and cerebrovascular systems. As yet, to the authors’ knowledge, only one study has been published on hair testosterone in eugonadal and hypogonadal men^28^. In this study by Thompson *et al*., the difference in serum testosterone between the three groups (eugonadal, untreated hypogonadal and treated hypogonadal) is evident, but, no significant differences in hair testosterone concentrations were found between eugonadal and untreated hypogonadal men. This raises the question whether some men might have been misclassified as hypogonadal as only one blood sample was analysed. If a method could be developed to reliably discern between untreated hypogonadal and eugonadal men, it would substantially facilitate diagnosis and monitoring of treatment. Future studies aiming at validating the physiological relevance of hair testosterone, exploring the temporal and segmental intra- and interindividual variability, will ultimately reveal the true potential of hair analysis as a complement to testosterone measured in blood or saliva.

The lack of selectivity for testosterone of our RIA may be seen as limitation. However, it has from the outset been our intention to measure testosterone and its analogs with a low detection limit in small hair samples in order to measure the testosterone-like immunoreactivity in the hair of both men and women. Our hypothesis is that the measurement of testosterone-like immunoreactivity in hair may be of even greater diagnostic relevance than the selective measurement of testosterone only. We expect that there are numerous factors that influence the concentrations of androgens in hair, in a similar way that hair cortisol concentrations have been shown to correlate with, for example, age, ethnicity and some anthropometric measures^29,30^. Thus, if other variables affecting the androgenic hair hormone concentrations would be found and accounted for, it is possible that the differences in androgenic hair hormone concentrations between males and females would be more pronounced.

In conclusion, we have developed an extraction method and in-house RIA for measuring testosterone-like immunoreactivity in the hair of both men and women, using hair samples weighing only 5 to 10 mg. Extraction of non-pulverised hair decreased the precision of the measurement, causing a poorer separation in the testosterone-like immunoreactivity between the sexes. This is evidence that pulverising the hair prior to hormone extraction is advantageous for hormone analysis in hair.
